# Hypothalamic–Pituitary–Adrenal Axis Dysfunction Elevates SUDEP Risk in a Sex-Specific Manner

**DOI:** 10.1523/ENEURO.0162-24.2024

**Published:** 2024-07-09

**Authors:** Trina Basu, Pantelis Antonoudiou, Grant L. Weiss, Emanuel M. Coleman, Julian David, Daniel Friedman, Juliana Laze, Misty M. Strain, Orrin Devinsky, Carie R. Boychuk, Jamie Maguire

**Affiliations:** ^1^Tufts University School of Medicine, Boston, Massachusetts 02111; ^2^University of Missouri, Columbia, Missouri 65211; ^3^New York University Langone Medical Center Comprehensive Epilepsy Center, New York, New York 10016

**Keywords:** comorbidities, epilepsy, HPA axis, neuroendocrine, SUDEP

## Abstract

Epilepsy is often comorbid with psychiatric illnesses, including anxiety and depression. Despite the high incidence of psychiatric comorbidities in people with epilepsy, few studies address the underlying mechanisms. Stress can trigger epilepsy and depression. Evidence from human and animal studies supports that hypothalamic–pituitary–adrenal (HPA) axis dysfunction may contribute to both disorders and their comorbidity ( Kanner, 2003). Here, we investigate if HPA axis dysfunction may influence epilepsy outcomes and psychiatric comorbidities. We generated a novel mouse model (*Kcc2*/*Crh* KO mice) lacking the K^+^/Cl^−^ cotransporter, KCC2, in corticotropin-releasing hormone (CRH) neurons, which exhibit stress- and seizure-induced HPA axis hyperactivation ( Melon et al., 2018). We used the *Kcc2*/*Crh* KO mice to examine the impact on epilepsy outcomes, including seizure frequency/burden, comorbid behavioral deficits, and sudden unexpected death in epilepsy (SUDEP) risk. We found sex differences in HPA axis dysfunction’s effect on chronically epileptic KCC2/Crh KO mice seizure burden, vulnerability to comorbid behavioral deficits, and SUDEP. Suppressing HPA axis hyperexcitability in this model using pharmacological or chemogenetic approaches decreased SUDEP incidence, suggesting that HPA axis dysfunction may contribute to SUDEP. Altered neuroendocrine markers were present in SUDEP cases compared with people with epilepsy or individuals without epilepsy. Together, these findings implicate HPA axis dysfunction in the pathophysiological mechanisms contributing to psychiatric comorbidities in epilepsy and SUDEP.

## Significance Statement

Our work provides new insight into a potential novel pathophysiological mechanism contributing to psychiatric illnesses and sudden unexpected death in epilepsy (SUDEP) in epilepsy patients, implicating hypothalamic–pituitary–adrenal (HPA) axis dysfunction in negative outcomes associated with epilepsy. This study is the first to link HPA axis dysfunction to SUDEP risk. Chronically epileptic male mice with exaggerated HPA axis dysfunction had increased SUDEP incidence. The translational relevance of these findings is supported by neuroendocrine abnormalities observed in postmortem samples from individuals that died of SUDEP. These data suggest that neuroendocrine mechanisms should be further explored in psychiatric comorbidities in epilepsy and SUDEP risk. Furthermore, neuroendocrine markers may be a biomarker for SUDEP risk.

## Introduction

Psychiatric comorbidities are highly prevalent in people with epilepsy (PWE), affecting ∼75%, with depression (55%) and anxiety (25–50%) being the most common (Kanner, 2003, 2006; Brandt et al., 2010). A cardinal feature of depression, the psychiatric disorder most commonly diagnosed in PWE, is hypothalamic–pituitary–adrenal (HPA) axis hyperactivity (Pariante and Lightman, 2008). The HPA axis mediates the body’s physiological response to stress, a major risk factor for depression and anxiety and a seizure trigger in many PWE (Sawyer and Escayg, 2010). Psychological or physiological stress induces an HPA-mediated neuroendocrine response, governed by corticotropin-releasing hormone (CRH) neurons in the paraventricular nucleus (PVN) of the hypothalamus. In response to stress or seizures, CRH is released and sequentially triggers the release of adrenocorticotropin hormone from the anterior pituitary gland and cortisol (CORT) from the adrenal glands (corticosterone in mice). Most PWE report stress as a trigger for seizures, and CORT levels are basally elevated in PWE and increase postictally, correlating with seizure severity (Sawyer and Escayg, 2010). Seizures activate the HPA axis in rodents (O'Toole et al., 2014). Stress hormones are proconvulsants; exacerbate neuropathology, comorbid behavioral deficits, and disease progression; and accelerate epileptogenesis (O'Toole et al., 2014; Wulsin et al., 2016, 2021; Hooper et al., 2018). Thus, we propose that HPA axis dysfunction may negatively impact epilepsy outcomes, including psychiatric comorbidities.

Here, we examined a genetic mouse model exhibiting HPA axis hyperactivation, epilepsy, and psychiatric disorders. The HPA axis is tightly regulated by GABAergic control of CRH neurons in the PVN. Stress- and seizure- induced HPA axis activation is driven by a collapse in the chloride gradient in CRH neurons, required for GABAergic inhibition and maintained by the K^+^/Cl^−^ cotransporter, KCC2 (O'Toole et al., 2014). To investigate HPA axis dysfunction in epilepsy and associated psychiatric comorbidities, we generated mice that lack KCC2 in CRH neurons (*Kcc2*/*Crh* KO; Melon et al., 2018). Mice with HPA axis hyperexcitability (*Kcc2*/*Crh* KO mice) exhibit an exaggerated seizure-induced HPA axis activation and increased vulnerability to anxiety- and depression-like behaviors associated with epilepsy and to sudden unexpected death in epilepsy (SUDEP). We demonstrate that pharmacological or chemogenetic attenuation of seizure-induced activation of the HPA axis reduces seizure burden and SUDEP incidence, further implicating HPA axis dysfunction in SUDEP. Our work suggests that HPA axis dysfunction may increase risk for SUDEP and that the *Kcc2*/*Crh* KO mouse model is a useful tool for studying the mechanisms contributing to SUDEP. Postmortem blood samples obtained from PWE and PWE with suspected SUDEP indicate that HPA axis dysfunction may contribute to SUDEP and suggest that neuroendocrine markers of HPA axis dysfunction may serve as novel biomarkers for those at risk for SUDEP.

## Materials and Methods

We studied adult (8–12 weeks) male and female Cre^−/−^ (WT) and *Kcc2*/*Crh* KO mice that we generated and characterized (Melon et al., 2018). The ventral intrahippocampal kainic acid (vIHKA) model was used to generate chronically epileptic mice (Zeidler et al., 2018). Some mice were bilaterally injected with AAV-hSyn-DIO-hM4D(Gi)-mCherry (Gi DREADD) in the PVN to inhibit CRH neuron activity and HPA axis activity. Some mice were implanted with a 10 mg, 21 day, slow-release RU486 pellet (Innovative Research of America, catalog #X-999). Electroencephalogram (EEG) recordings (24/7) were acquired as described (Hooper et al., 2018), and seizures were detected using a custom, in-house seizure detection app (Extended Data) and SUDEP was confirmed by EEG. Comorbid behavioral deficits were measured using the open-field (OF) test, light/dark (LD) box, forced swim test (FST), sucrose preference test (SPT), and the nestlet-shredding test (NST; Melon et al., 2018; Extended Data). Statistical tests were performed using GraphPad Prism 9. All data are represented as the mean ± SEM. All *p* values <0.05 were considered significant. *p < 0.05; **p < 0.01; ***p < 0.001; ****p < 0.0001. All statistics details are provided in Extended Data. Additional details regarding the methods are provided in Extended Data.

## Results

To examine the impact of HPA axis hyperexcitability on epilepsy, we used a mouse model that exhibits exacerbated seizure-induced activation of the HPA axis (*Kcc2*/*Crh* KO mice) and evaluated the impact on several epilepsy outcome measures, including spontaneous recurrent seizure frequency, characteristic neuropathological features, and psychiatric comorbidities.

### Seizure-induced activation of the HPA axis in *Kcc2*/*Crh* KO male mice

**Figure 1.**
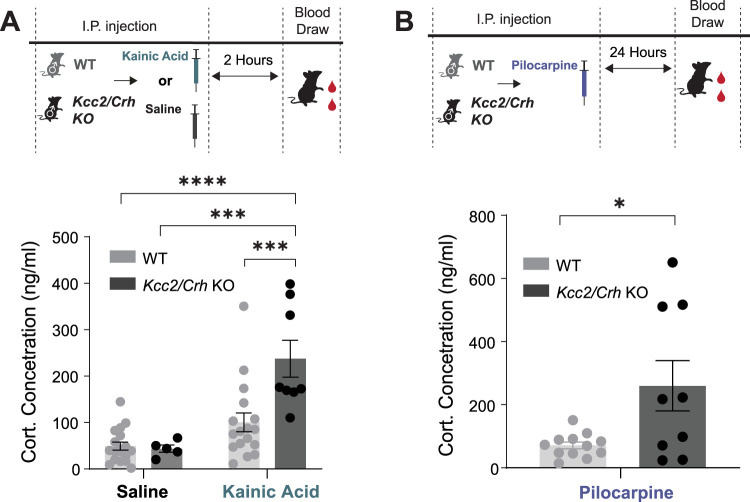
*Kcc2*/*Crh* KO male mice exhibit an exaggerated, seizure-induced activation of the HPA axis. ***A***, Circulating corticosterone concentration collected 2 h after intraperitoneal injections of either saline or KA in WT and *Kcc2*/*Crh* KO male mice. ***B***, Circulating corticosterone levels were quantified from serum collected 24 h following pilocarpine-induced SE in WT and *Kcc2*/*Crh* KO male mice. *n* = 18 (WT saline); 5 (*Kcc2*/*Crh* KO saline); 17 (WT KA); 8 (*Kcc2*/*Crh* KO KA); 12 (WT pilocarpine); 9 (*Kcc2*/*Crh* KO pilocarpine). Error bars represent ±SEM.

*Kcc2*/*Crh* KO male mice exhibit increased circulating levels of corticosterone following seizures induced with kainic acid (237.52 ± 39.66 ng/ml) or pilocarpine (259.8 ± 79.69 ng/ml) compared with WT (kainic acid, 100.48 ± 20.148 ng/ml; pilocarpine, 71.07 ± 10.85), which is significantly elevated compared with saline controls (49.01 ± 8.58 ng/ml; Fig. 1*A*,*B*). This initial pilot study focused only on male mice and was the motivation for a comprehensive investigation into the impact of HPA axis hyperexcitability on epilepsy outcomes in both male and female mice.

### Chronically epileptic male *Kcc2*/*Crh* KO mice exhibit increased vulnerability to negative affective states

**Figure 2.**
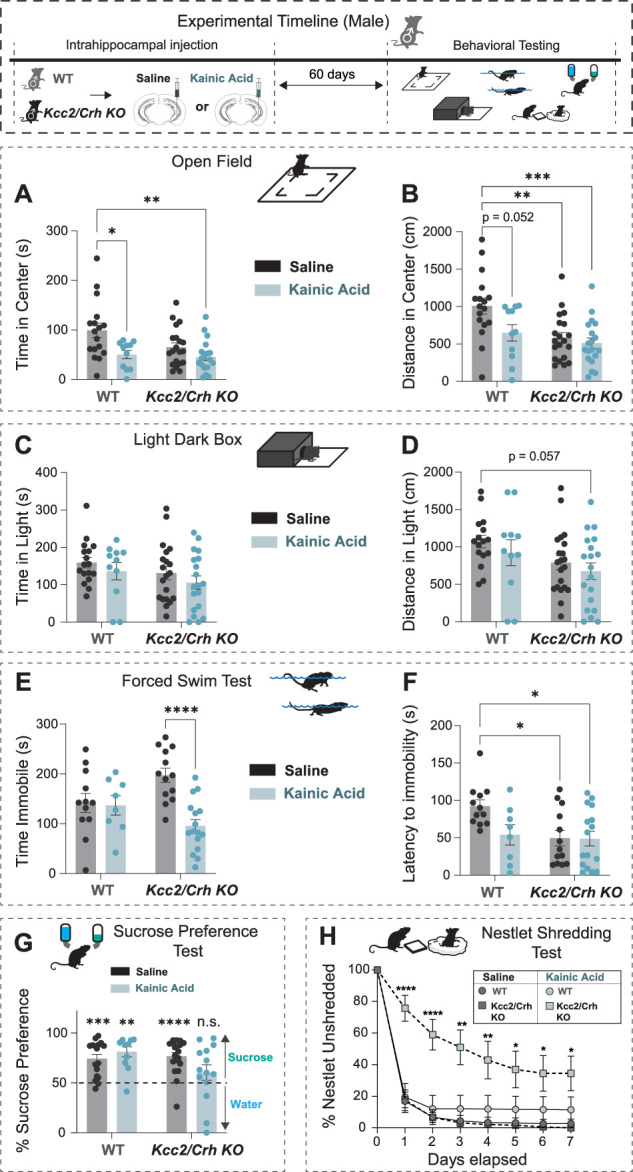
Chronically epileptic *Kcc2*/*Crh* KO male mice exhibit increased vulnerability to negative affective states compared with WT control mice. Top, Experimental paradigm illustrating the timeline of intrahippocampal injections and behavioral testing. Tests for avoidance (***A–D***), learned helplessness (***E***, ***F***), anhedonia (***G***), and goal-directed (***H***) behaviors were tested in control and chronically epileptic male WT and *Kcc2*/*Crh* KO mice. ***A***, ***B***, The average total time spent in the center (***A***) and total distance traveled in the center of the OF test (***B***). ***C***, ***D***, The average total time spent (***C***) and total distance traveled (***D***) in the light arena of the LD setup. ***E***, ***F***, The histograms depict the average total time spent immobile (***E***) and the latency to the first bout of immobility (***F***) in the FST. ***G***, The average percent sucrose preference measured over the course of a 7 d SPT paradigm. ***H***, The average percentage of unshredded nestlet material was weighed daily for 1 week. *N *= 11–20 per group. Error bars represent SEM. OF, open field; LD, light/dark; FST, forced swim test; SPT, sucrose preference test; NST, nestlet-shredding test. The proportion of chronically epileptic *Kcc2*/*Crh* KO mice that exhibit increased vulnerability to negative affective states compared with chronically epileptic (vIHKA) WT mice is provided in Extended Data Figure 2-1.

To examine the role of HPA axis dysfunction in comorbid psychiatric illnesses and epilepsy, we utilized *Kcc2*/*Crh* KO mice with HPA axis hyperexcitability and assessed behavioral deficits in chronically epileptic mice. In males, saline-injected *Kcc2*/*Crh* KO mice, chronically epileptic WT, and chronically epileptic mice *Kcc2*/*Crh* KO avoid spending time in the center of the OF arena compared with saline-injected WT mice (Fig. 2*A*,*B*), indicating that either kainic acid treatment or HPA axis hyperexcitability promotes avoidance behaviors in male mice. Chronically epileptic *Kcc2*/*Crh* KO mice also travel less cumulative distance in the light chamber of the LD box, another test of avoidance behavior (Fig. 2*D*), suggesting that HPA axis hyperexcitability may exacerbate aversion in chronically epileptic mice. Interestingly, chronically epileptic Cre^−/−^ (WT) mice do not exhibit deficits in stress-induced helplessness and hedonic behaviors as assessed by the FST and SPT, respectively (Fig. 2*E*–*G*), which differs from the phenotype observed in chronically epileptic C57Bl/6J mice (Colmers et al., 2023). Chronically epileptic *Kcc2*/*Crh* KO mice exhibit increased total time spent immobile and both saline-injected and chronically epileptic *Kcc2*/*Crh* KO exhibit a decreased latency to immobility in the FST compared with saline-injected WT mice (Fig. 2*E,F*), indicating that either kainic acid treatment or HPA axis hyperexcitability promotes stress-induced helplessness. Chronically epileptic *Kcc2*/*Crh* KO male mice also exhibit decreased sucrose preference compared with saline-injected WT, saline-injected *Kcc2*/*Crh* KO, and chronically epileptic WT mice (Fig. 2*G*). Furthermore, we found that chronically epileptic *Kcc2*/*Crh* KO mice exhibit severe deficits in the NST compared with either saline-injected WT, saline-injected *Kcc2*/*Crh* KO, or chronically epileptic WT mice (Fig. 2*H*, Extended Data Table 2-1). The NST has been used to assess spontaneous, goal-directed behaviors as a measure of motivation (Palmiter, 2008), features related to psychiatric illnesses (Dorninger et al., 2020), including anxiety-like behaviors (Li et al., 2006). Collectively, these data support that HPA axis dysfunction contributes to behavioral deficits associated with chronic epilepsy.

10.1523/ENEURO.0162-24.2024.f2-1 Figure 2-1 A greater proportion of the chronically epileptic Kcc2/Crh KO mouse population exhibit increased vulnerability to negative affective states compared to chronically epileptic (vIHKA) WT mice. Smoothed population distributions (top) along with raw histogram distribution (bottom) of performance in the Open Field (A-B), Light Dark box (C-D), and Forced Swim Test (E-F) between chronically epileptic WT (left) and Kcc2/Crh KO (right) mice. In each smoothed plot, the lighter color represents the underperforming, more vulnerable population while the black distribution plots represent the resilient groups. # denotes instances where only one peak was detected, so population distributions were delineated by the mean of the data. Download Figure 2-1, TIF file.

We analyzed the distribution of the populations for the chronically epileptic WT and *Kcc2*/*Crh* KO mice using a kernel density estimate to determine a continuous probability density curve where the population was delineated by peaks into either putative vulnerable or resilient groups. Using this algorithm, we found that compared with chronically epileptic WT mice, a greater proportion of the chronically epileptic *Kcc2*/*Crh* KO mice were vulnerable across the behavioral paradigms (Extended Data Fig. 2-1). These data suggest that in male *Kcc2*/*Crh* KO mice, HPA axis dysfunction may increase vulnerability to negative affective states associated with chronic epilepsy.

### Chronically epileptic female *Kcc2*/*Crh* KO mice display increased negative affective states

**Figure 3.**
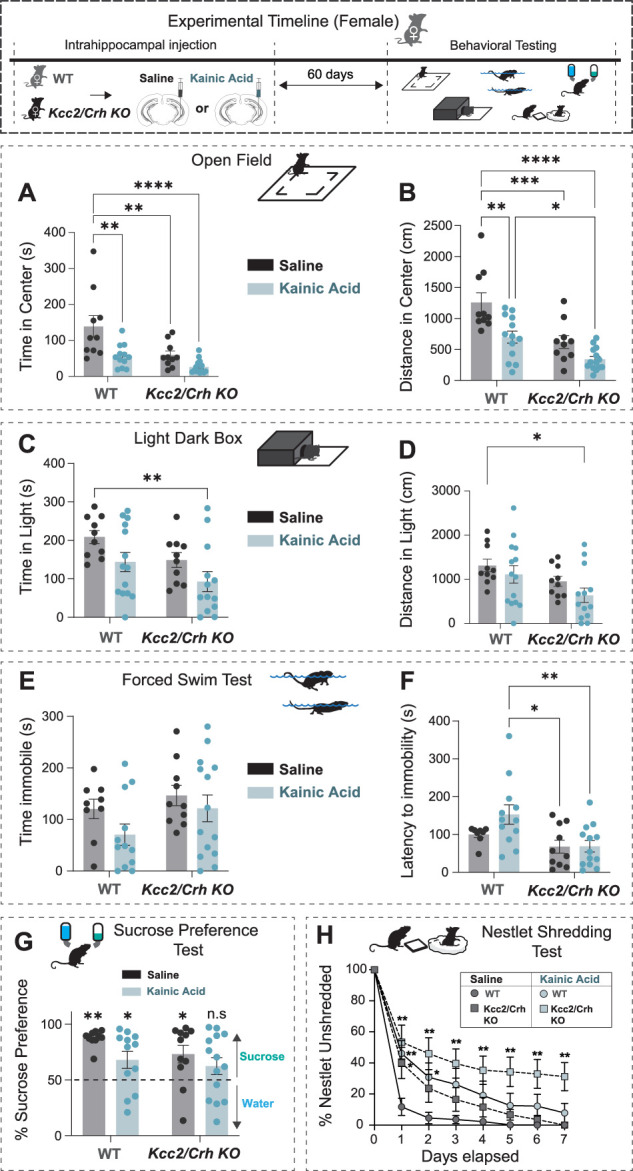
Chronically epileptic females with HPA axis dysfunction exhibit increased incidence of negative affective states. Top, Experimental paradigm illustrating the timeline of intrahippocampal injections and behavioral testing. Tests for avoidance (***A–D***), learned helplessness (***E***, ***F***), anhedonia (***G***), and goal-directed (***H***) behaviors were tested in control and chronically epileptic female WT and *Kcc2*/*Crh* KO mice. ***A***, ***B***, The total amount of the time spent in the center of the OF (***A***) and the total distance traveled in the OF (***B***) were measured in the control and chronically epileptic female WT and *Kcc2*/*Crh* KO mice. ***C***, ***D***, The total time spent (***C***) and the total distance traveled (***D***) in the light arena of the LD box was measured and quantified. ***E***, ***F***, The total time spent immobile (***E***) and the latency to immobility (***F***) in a 6 min FST paradigm were measured and quantified. ***G***, The average sucrose water consumed over a 7 d period was measured for the SPT paradigm. ***H***, Chronically epileptic and control WT and *Kcc2*/*Crh* KO mice were singly housed and given a piece of unshredded nestlet. The percentage of nestlet that remained unshredded was weighed daily over the course of 7 d. *N *= 10–14 mice per group. Error bars represent SEM. OF, open field; LD, light/dark; FST, forced swim test; SPT, sucrose preference test; NST, nestlet-shredding test.

Compared with saline-injected WT female mice, chronically epileptic WT and *Kcc2*/*Crh* KO female mice exhibit increased avoidance behaviors in the OF paradigm, spending significantly less time and traveling less distance in the center (Fig. 3*A*,*B*). Chronically epileptic *Kcc2*/*Crh* KO mice spend less time in the center than chronically epileptic WT mice (Fig. 3*B*). In the LD box paradigm, we find that only chronically epileptic female *Kcc2*/*Crh* KO mice avoid the light box more than the saline-injected WT female mice (Fig. 3*C*,*D*). These data indicate that with greater HPA axis dysfunction, there is an additive worsening of avoidance behavior in chronically epileptic mice.

As in the male mice, chronically epileptic WT female mice did not exhibit robust deficits in stress-induced helplessness and hedonic behaviors as assessed by the FST and SPT, respectively (Fig. 3*E*–*G*). However, chronically epileptic WT mice display greater variability in the both FST and sucrose preference compared with saline-injected WT mice (Fig. 3*E*–*G*). Chronically epileptic *Kcc2*/*Crh* KO female mice exhibit decreased preference for sucrose in the SPT compared with saline-injected WT, saline-injected *Kcc2*/*Crh* KO, and chronically epileptic WT mice (Fig. 3*G*), suggesting that HPA axis dysfunction negatively impacts hedonic behavior in chronic epilepsy. In addition, chronically epileptic *Kcc2*/*Crh* KO female mice have decreased latency to immobility in the FST compared with chronically epileptic WT mice (Fig. 3*F*). Chronically epileptic *Kcc2*/*Crh* KO mice also exhibit a significant deficit in NST across all days tested (Fig. 3*H*; Extended Data Table 3-1), consistent with HPA axis dysfunction-mediated worsening of negative affective states in chronically epileptic female mice. In summary, our data shows that HPA axis dysfunction differentially impacts negative affective states in chronically epileptic male and female mice.

### HPA axis hyperexcitability exaggerates neuropathological features of chronic epilepsy

Hallmark neuropathological features of temporal lobe epilepsy, including hippocampal mossy fiber sprouting (MFS) and dentate granule cell dispersion (DGCD), have been well characterized in both preclinical models of epilepsy and in PWE. Chronic pathological activation of the HPA axis in clinical models of mood disorders have also been shown to compromise hippocampal function and integrity (de Kloet et al., 2005; Krugers et al., 2010; McEwen, 2016; McEwen et al., 2016; McEwen and Magarinos, 2017). Here, we assessed the impact of HPA axis hyperexcitability on the neuropathological features of epilepsy. Eight weeks following intrahippocampal injection of either kainic acid or saline, brains from WT and *Kcc2*/*Crh* KO mice were collected, processed, and stained with either ZnT3 (for MFS) or DAPI and NeuN (for DGCD; Extended Data Figs. 4-2, 5-1). We found a pronounced effect of kainic acid injection in MFS in WT and *Kcc2*/*Crh* KO mice, with *Kcc2*/*Crh* KO male mice exhibiting increased MFS compared with chronically epileptic WT male mice (Extended Data Fig. 4-2) and *Kcc2*/*Crh* KO female mice exhibiting decreased MFS compared with chronically epileptic WT female mice (Extended Data Fig. 5-1*A*,*B*). The finding that HPA axis dysfunction appears to have a differential impact on MFS by sex suggests that exaggerated HPA axis dysfunction may specifically protect against MFS in chronically epileptic females, but not males. Additionally, chronically epileptic male and female WT and *Kcc2*/*Crh* KO mice display DGCD in the kainic acid-injected hemisphere compared with the contralateral, saline-injected hemisphere, as indicated by reduced proximity to neighboring cells and cell density (Extended Data Figs. 4-2, 5-1*C*–*F*). However, we did not observe any differences in the DGCD measures between the chronically epileptic WT and *Kcc2*/*Crh* KO mice. Our data suggests that HPA axis dysfunction affects MFS but has no impact on DGCD.

### HPA axis dysfunction increases SUDEP incidence in chronically epileptic male but not female mice

**Figure 4.**
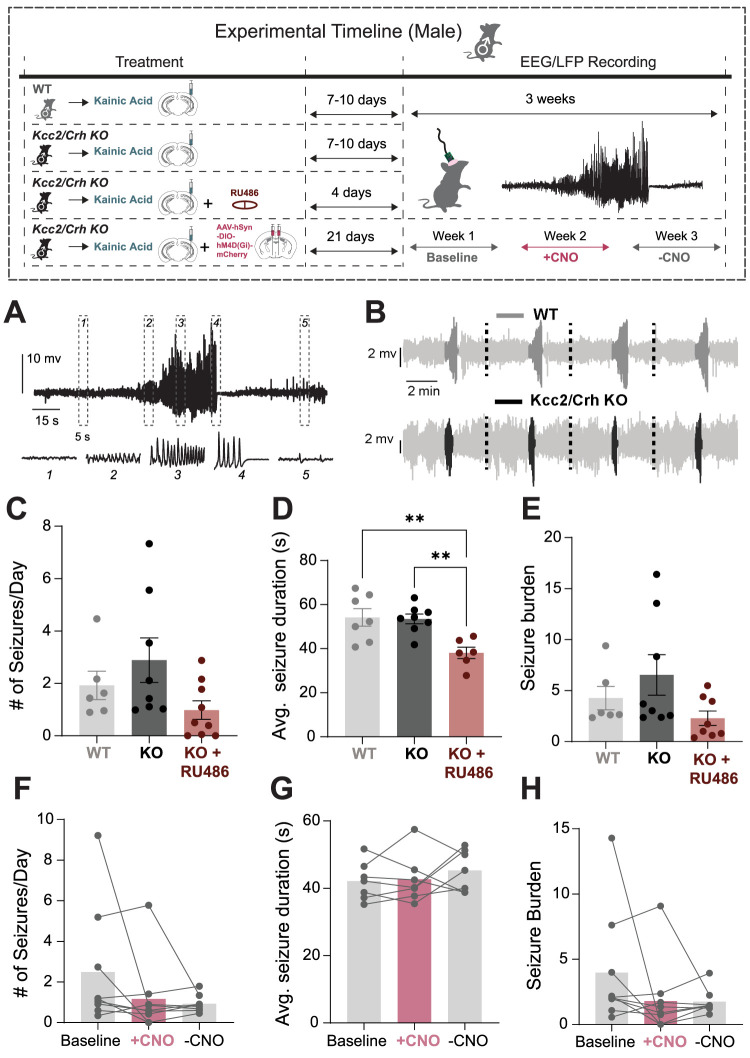
HPA axis dysfunction does not worsen spontaneous seizure activity in chronic epilepsy in males. Top, Experimental paradigm illustrating the timeline of intrahippocampal injections with treatment and EEG/LFP recordings. The seizure detection pipeline is described in Extended Data Figure 4-1. ***A***, Representative recorded seizure from hippocampal local field potential. Orange squares indicate time magnified 5 s traces. ***B***, Example detected hippocampal seizures which were concatenated from a WT and a *Kcc2*/*Crh* KO (KO) mouse. ***C–E***, Mean (±SEM) number of daily seizure occurrences (***C***), seizure duration (***D***), and seizure burden (***E***) for chronically epileptic WT, *Kcc2*/*Crh* KO, and *Kcc2*/*Crh* KO mice treated with RU486, a glucocorticoid receptor antagonist. ***F–H***, Average number of daily seizures (***F***), seizure durations (***G***), and seizure burden (***H***) for chronically epileptic *Kcc2*/*Crh* KO mice bilaterally injected with hM4D(Gi)-DREADDs in the PVN. Graphs ***F–H*** depict mean seizure activity collected via EEG recordings that were made prior to CNO administration (baseline), during CNO administration (+CNO), and during a week free of CNO administration (−CNO). Seizure burden was calculated by multiplying the total number of seizures a mouse exhibited by their average seizure duration and dividing this value by the total EEG recording hours. Error bars represent SEM. The impact of HPA axis dysfunction on neuropathological features of epilepsy in males is provided in Extended Data Figure 4-2.

Spontaneous recurrent seizure frequency was measured using 24/7 EEG recording in chronically epileptic WT and *Kcc2*/*Crh* KO mice. There were no significant differences in the number of daily seizures, average seizure duration, or seizure burden in WT versus *Kcc2*/*Crh* KO male mice (Fig. 4*A*–*E*). Remarkably, we found that 38.7% of *Kcc2*/*Crh* KO male mice died of EEG confirmed SUDEP within 26 d post status epilepticus (SE) with no deaths observed in chronically epileptic WT male mice (Fig. 6*A*). This unexpected discovery potentially links HPA axis dysfunction to SUDEP risk.

10.1523/ENEURO.0162-24.2024.f4-1 Figure 4-1 Seizure detection pipeline. (A) Left - Datasets used to train and test the seizure detection algorithm, Upper Right – Example traces for no-seizure and seizure (5 second periods), Bottom Right – Minimum number of seizure segments for an event to qualify as a seizure. (B) Histograms with KDE plots showing the Top – number of false detected seizures per hour and Bottom – percentage of detected seizures; Pink line denotes performance of chosen method. (C) Scatterplot of percent detected seizures vs false positive rate. Pink dot indicates performance of chosen method. Detailed pipelines can be found at https://github.com/neurosimata/seizy. Download Figure 4-1, TIF file.

10.1523/ENEURO.0162-24.2024.f4-2 Figure 4-2 HPA axis dysfunction worsens MFS in male chronically epileptic mice. (A) Representative coronal sections of the hippocampus collected from control and chronically epileptic adult, male WT and Kcc2/Crh KO mice and stained with ZnT3 to quantify MFS. White arrow indicates mossy fiber sprouting. In some slices, we observed that dentate completely lost structural integrity as assessed by ZnT3 staining; example indicated by a red outline (B) The mean (±SEM) percent change in MFS was quantified in the ipsilateral hemisphere and normalized to the mean percent change in MFS of the contralateral hemisphere of chronically epileptic WT and Kcc2/Crh KO mice. Dotted black line indicates no MFS in animals that received saline injection. In slices with complete loss of structural integrity, MFS was quantified as the full dentate length. Those slices are indicated by colored filled dots on the graph. (C-D) Representative sections stained with NeuN to visualize DGCD in WT (C) and Kcc2/Crh mice (D). Pink outlines were automatically generated through Cell Profiler and indicate cells where NeuN and DAPI colocalize. (E) The mean (±SEM) number of adjoining neighboring neuronal cells was quantified for the ipsilateral hemisphere and normalized to the mean number of immediate neighboring neuronal cells on the contralateral hemisphere for both control and chronically epileptic WT and Kcc2/Crh KO mice. (F) The total number of cells within the manually defined dentate gyrus area was quantified on the ipsilateral hippocampal hemisphere and normalized to the cell density of the non-injected, contralateral hippocampal hemisphere. n = brain slice sections. Error bars represent ± SEM. WT, wild type; Sal, saline; KA, kainic acid; DGCD, dentate granule cell dispersion; Norm, normalized. Download Figure 4-2, TIF file.

**Figure 5.**
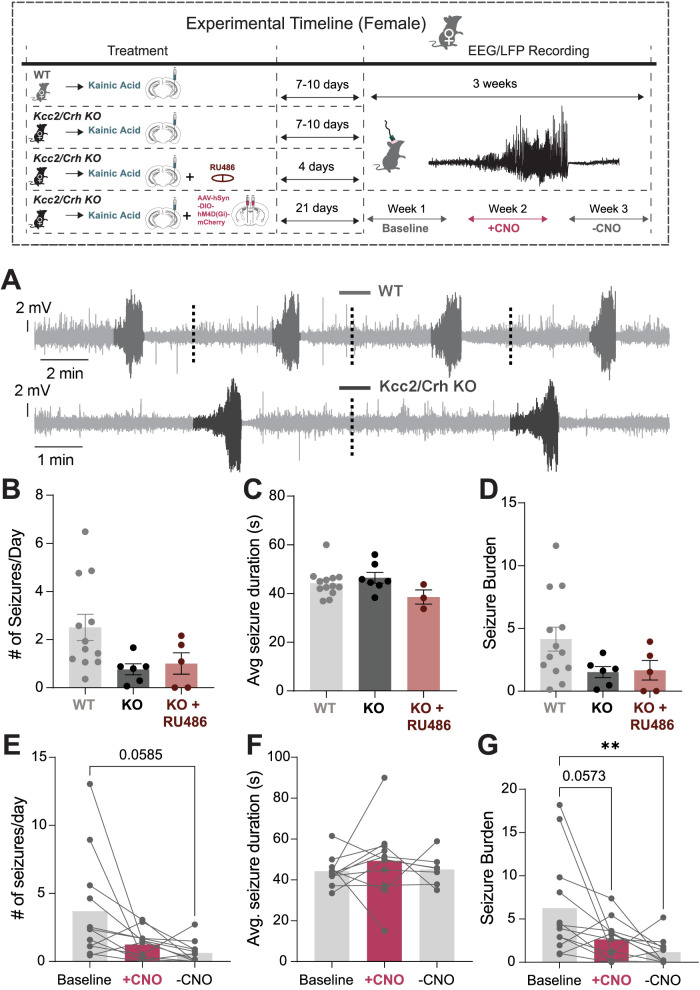
HPA dysfunction does not alter seizure severity in chronically epileptic female *Kcc2*/*Crh* KO mice. Top, Experimental paradigm illustrating the timeline of intrahippocampal injections with treatment and EEG/LFP recordings. ***A***, A representative EEG trace showing concatenated seizure events detected from WT and *Kcc2*/*Crh* KO female mice recorded from hippocampal local field potential. ***B–D***, The seizure frequency (***B***), average seizure duration (***C***), and overall seizure burden (***D***) quantified for chronically epileptic female WT, *Kcc2*/*Crh* KO, and *Kcc2*/*Crh* KO mice treated with the glucocorticoid receptor antagonist, RU486. ***F–H***, The daily seizure frequency (***E***), mean seizure duration (***F***), and overall seizure burden (***G***) quantified from chemogenetically manipulated chronically epileptic female *Kcc2*/*Crh* KO mice. Each period (baseline, with CNO, and without CNO treatment) was measured over a 7 d period. Seizure burden was calculated by multiplying the total number of seizures a mouse exhibited by their average seizure duration and dividing this value by the total EEG recording hours. Error bars represent SEM. The impact of HPA axis dysfunction on neuropathological features of epilepsy in females is provided in Extended Data Figure 5-1.

**Figure 6.**
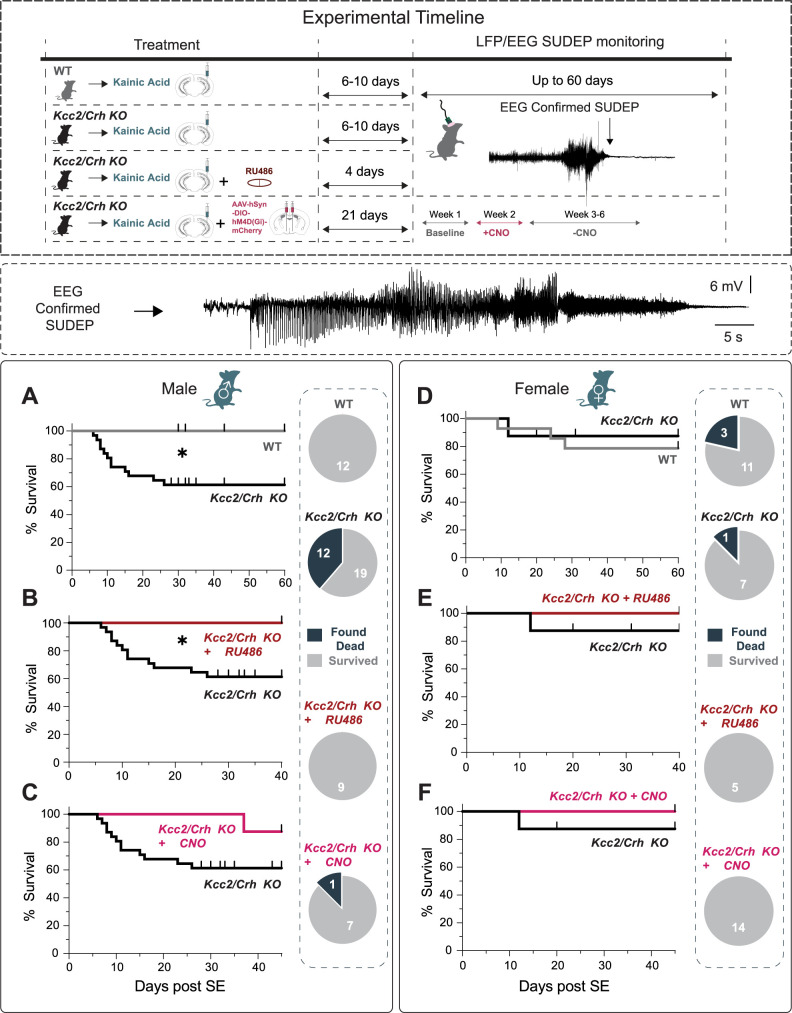
HPA axis dysfunction in chronically epileptic *Kcc2*/*Crh* KO mice increases SUDEP incidence in males. Top, Experimental paradigm illustrating the timeline of intrahippocampal injections with treatment and EEG/LFP recordings for SUDEP monitoring. ***A–C***, Percent survival following vIHKA-induced SE in chronically epileptic WT and *Kcc2*/*Crh* KO male mice (***A***), in chronically epileptic *Kcc2*/*Crh* KO mice treated with the glucocorticoid receptor antagonist, RU486 (***B***), and in chronically epileptic *Kcc2*/*Crh* KO mice expressing Gi DREADDs and given CNO (***C***). SUDEP, sudden unexpected death in epilepsy; WT, wild-type; SE, status epilepticus. ***D–F***, Percent survival after kainic acid-induced SE in chronically epileptic female WT and *Kcc2*/*Crh* KO female mice (***D***), in chronically epileptic female *Kcc2*/*Crh* KO mice treated with and without the glucocorticoid receptor antagonist, RU486 (***E***), and in chronically epileptic *Kcc2*/*Crh* KO mice treated with Gi DREADDs and given CNO via drinking water to reduce HPA axis activity (***F***). Upward ticks on survival plots indicate the time that mice were killed. For visual comparisons, the Kcc2/Crh survival plots have been replotted in each subpanel (same dataset) across male (***A–C***) and across female (***D–F***) mice.

Chronically epileptic female *Kcc2*/*Crh* KO mice had a lower seizure frequency average when compared with WT mice, although this effect was not statistically significant, probably due to the large variability in WT mice (Fig. 5*A*–*D*; WT = 2.81 ± 0.605 seizures/day vs *Kcc2*/*Crh* KO = 0.82 ± 0.207 seizures/day). In contrast to males, chronically epileptic female *Kcc2*/*Crh* KO mice do not exhibit an increase in SUDEP incidence at levels seen in the chronically epileptic male *Kcc2*/*Crh* KO mice (Fig. 6*A*).

10.1523/ENEURO.0162-24.2024.f5-1 Figure 5-1 HPA axis dysfunction worsens MFS in female chronically epileptic mice. (A) Representative coronal sections of the hippocampus collected from control and chronically epileptic adult, female WT and Kcc2/Crh KO mice and stained with ZnT3 to quantify MFS. White arrows indicate MFS. (B) The mean (±SEM) percent change in MFS was quantified in the ipsilateral hemisphere and normalized to the mean percent change in MFS of the contralateral hemisphere of chronically epileptic WT and Kcc2/Crh KO mice. Dotted black line indicates no MFS in animals that received saline injection. Representative sections stained with NeuN to visualize DGCD in WT (C) and Kcc2/Crh KO mice (D). Pink outlines were automatically generated through Cell Profiler and indicate cells where NeuN and DAPI colocalize. (E) The mean (±SEM) number of adjoining neighboring neuronal cells was quantified for the ipsilateral hemisphere and normalized to the mean number of immediate neighboring neuronal cells on the contralateral hemisphere for both control and chronically epileptic WT and Kcc2/Crh KO female mice. (F) The total number of cells within the manually defined dentate gyrus area was quantified on the ipsilateral hippocampal hemisphere and normalized to the cell density of the non-injected, contralateral hippocampal hemisphere. n = brain slice sections. Error bars represent ± SEM. WT, wild type; Sal, saline; KA, kainic acid; DGCD, dentate granule cell dispersion; Norm, normalized. Download Figure 5-1, TIF file.

To interrogate whether HPA axis dysfunction in the *Kcc2*/*Crh* KO male mice contributes to the increased risk of SUDEP, we pharmacologically blocked glucocorticoid signaling using RU486, a 21 d slow-release pellet that was implanted during kainic injection (Fig. 4, timeline). Pharmacological inhibition of glucocorticoid signaling using RU486 did not alter seizure frequency (Fig. 4*C*) or seizure burden (Fig. 4*E*); however, it significantly reduced the average seizure duration compared with chronically epileptic WT and *Kcc2*/*Crh* KO mice (Fig. 4*D*). Importantly, RU486 treatment prevented the increased SUDEP incidence in chronically epileptic *Kcc2*/*Crh* KO mice, occurring in 0 of the 9 mice tested, compared with 12 out of 19 untreated chronically epileptic *Kcc2*/*Crh* KO mice (Fig. 6*B*). These data indicate that HPA axis dysfunction contributes to SUDEP risk in males, and attenuation of seizure-induced activation of the HPA axis can reduce SUDEP incidence.

Pharmacological inhibition of glucocorticoid signaling with RU486 in chronically epileptic female *Kcc2*/*Crh* KO mice did not significantly alter seizure properties when compared with untreated chronically epileptic *Kcc2*/*Crh* KO female mice. We did not observe a difference in daily seizure frequency (Fig. 5*B*), average seizure duration (Fig. 5*C*), or overall seizure burden (Fig. 5*D*) in chronically epileptic female *Kcc2*/*Crh* KO mice treated with RU486. No difference in SUDEP incidence was observed between chronically epileptic female *Kcc2*/*Crh* KO mice treated with or without RU486 (Fig. 6*E*).

To further examine whether exaggerated HPA axis dysfunction in the chronically epileptic *Kcc2*/*Crh* KO male mice contributes to the increased risk of SUDEP, we used chemogenetics to attenuate HPA axis activity by suppressing the activity of CRH neurons in the PVN of the hypothalamus which govern HPA axis activity. Silencing the HPA axis by expressing Gi DREADDs in the PVN in male *Kcc2*/*Crh* KO mice and delivering the synthetic ligand, clozapine-N-oxide (CNO), via drinking water did not have any impact on overall seizure frequency, duration, or burden compared with the baseline (Fig. 4*F*–*H*) but substantially reduced SUDEP incidence, where only 1 out of 8 mice died of SUDEP compared with 12 out of 19 untreated chronically epileptic *Kcc2*/*Crh* KO mice, although this effect was not statistically significant (Fig. 6*C*). In fact, the one SUDEP incidence in the chronically epileptic male *Kcc2*/*Crh* KO group injected with Gi DREADD occurred during the week when CNO was removed. This further suggests that HPA axis dysfunction contributes to SUDEP and that regulating HPA axis activity can reduce the risk of SUDEP. Interestingly, in chronically epileptic female *Kcc2*/*Crh* KO mice, seizure frequency and seizure burden remained reduced in the post CNO off period (Fig. 5*E*,*G*), and treatment with Gi DREADDs resulted in no SUDEP incidence in the female *Kcc2*/*Crh* KO mice (Fig. 6*F*). Thus, in these chronically epileptic male and female mice, SUDEP incidence does not correlate with seizure severity outcomes. Overall, our data suggest that HPA axis dysfunction impacts epilepsy outcomes and SUDEP risk differently in male and female mice.

### Neuroendocrine dysfunction in epilepsy may increase SUDEP risk

**Figure 7.**
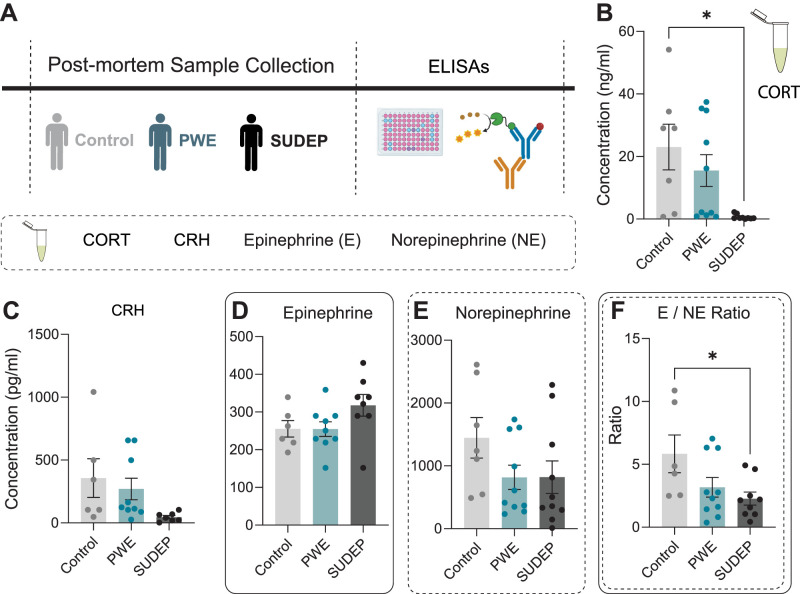
Chronic HPA axis dysfunction may increase SUDEP risk in PWE. ***A***, Experimental schematic. ***B***, Postmortem analysis of circulating CORT, (***C***) CRH, (***D***) epinephrine, (***E***) norepinephrine (NE), and (***F***) E/NE ratio in control subjects, PWE, and subjects who died of SUDEP. Error bars represent SEM. Data were analyzed using a one-way ANOVA. PWE, person(s) with epilepsy; SUDEP, sudden unexpected death in epilepsy.

Data from our preclinical model suggests that HPA axis dysfunction may increase susceptibility to SUDEP, a finding that is relevant to patients that may identify a biomarker for SUDEP risk. To understand whether our findings translate to the human condition, we ran enzyme-linked immunoassays for CRH, CORT, epinephrine, and norepinephrine in postmortem blood samples collected from PWE with or without suspected SUDEP compared with individuals with no history of epilepsy (samples obtained from the North American SUDEP Registry at New York University Langone Health). Contrary to what we hypothesized, we observed a significant decrease in CORT and CRH in PWE with suspected SUDEP compared with either PWE or individuals with no history of epilepsy (Fig. 7*A*–*C*). This effect was only statistically significant in CORT, probably due to the limited sample number and high variability (Fig. 7*C*). This substantial decrease in neuroendocrine stress mediators in the PWE with suspected SUDEP is consistent with HPA axis dysfunction contributing to SUDEP. However, there were no differences between the three groups in epinephrine (Fig. 7*D*) and norepinephrine levels (Fig. 7*E*), additional neuroendocrine mediators of stress, suggesting that the integrity of the samples is intact. However, the epinephrine/norepinephrine (E/NE) ratio was significantly decreased in PWE with suspected SUDEP compared with either PWE who died from other causes or individuals without epilepsy. This data supports neuroendocrine disruptions are associated with SUDEP.

## Discussion

Consistent with clinical reports of high incidence rates of anxiety and depression in PWE, several studies found that chronically epileptic mice exhibit increased anxiety- and depression-like behaviors (Gröticke et al., 2007, 2008; Muller et al., 2009; Hooper et al., 2018; Zeidler et al., 2018). Here, we show that mice with HPA axis dysfunction (*Kcc2*/*Crh* KO mice) have an increased predisposition to behavioral deficits associated with chronic epilepsy. While there are no significant differences in the aggregated behavioral outcomes between male chronically epileptic WT or *Kcc2*/*Crh* KO male mice, possibly reflecting a floor effect in behavioral severity likely due to activation of the HPA axis in both experimental groups. In fact, we demonstrate increased behavioral deficits induced by either kainic acid treatment or HPA axis hyperexcitability. There is, however, a high variability in behavioral outcomes, distributing into resilient and vulnerable populations in which there is an increased vulnerable population in epileptic mice with HPA axis dysfunction (*Kcc2*/*Crh* KO mice). Interestingly, the impact of HPA axis dysfunction on comorbid behavioral deficits in chronically epileptic mice is more pronounced in females compared with males, which is consistent with the evidence that there is an increased incidence of psychiatric comorbidities in women with epilepsy (Gaus et al., 2015; Zhu et al., 2022; Revdal et al., 2023).

This study makes the unexpected discovery that HPA axis hyperexcitability increases SUDEP risk. We demonstrate that chronically epileptic male mice with exaggerated seizure-induced activation of the HPA axis (*Kcc2*/*Crh* KO mice) exhibit increased mortality due to SUDEP, with nearly 40% succumbing to SUDEP. To our knowledge, this mouse model represents the first potential environmental link to SUDEP risk. In contrast, the SUDEP phenotype was not observed in female mice with exaggerated seizure-induced activation of the HPA axis. These findings are consistent with the increased incidence of SUDEP observed in men (Hesdorffer et al., 2011; Sveinsson et al., 2017).

We confirmed that the increased risk of SUDEP in this model is directly related to HPA axis dysfunction since pharmacological suppression of the HPA signaling prevents SUDEP incidence in this model. While chemogenetic suppression of the HPA signaling also reduced SUDEP, this effect did not reach statistical significance due to the death of one mouse following the cessation of CNO treatment. However, we were also concerned that the SUDEP phenotype may be an artifact of the mouse model with no translational relevance to the human condition. These concerns were dispelled by the evidence of neuroendocrine alterations in blood samples from PWE that died of suspected SUDEP compared with non-PWE or PWE (without suspected SUDEP) samples. Although we observed reduced CORT levels during active epilepsy in our acute preclinical models, these data together suggest there are neuroendocrine abnormalities associated with SUDEP in both preclinical models and postmortem samples. We speculate that the persistent seizure-induced overactivation of the HPA axis in chronic epilepsy leads to a collapse in the HPA axis over time, which in turn may substantially increase SUDEP risk. It should be noted that there are other potential variables in the postmortem human samples which may contribute to these differences, such as differences in the time of day (diurnal fluctuations in CORT) or time to sample collection, which may indirectly result from the fact that most SUDEP events occur at night (Devinsky et al., 2016). However, it has been shown that CORT levels are stable over the time of collection, at least in salivary CORT (Garde and Hansen, 2005), suggesting that this may not be a confounding factor. Furthermore, we demonstrate that the absolute levels of epinephrine and norepinephrine are not altered in PWE with SUDEP, suggesting that the integrity of the samples is intact. Thus, these data demonstrate that HPA axis dysfunction may be a novel mechanism contributing to SUDEP and is the first potential link to an environmental insult to be implicated in the mechanisms contributing to SUDEP.

Stress is linked to sudden death in people without epilepsy, primarily due to heart failure (Lampert, 2009). Between 20 and 40% of sudden cardiac deaths are precipitated by stress (Vlastelica, 2008). Stress increases arrhythmias linked to the increased risk of sudden death (Lampert, 2009). Although the mechanism through which stress increases the risk for sudden death is poorly understood, it likely involves the ability of stress to induce changes in autonomic function (Ginsberg, 2016). Similar to stress, seizures are also associated with cardiac changes, such as arrhythmias, suggesting that stress should be evaluated as a potential risk factor for SUDEP (Lathers and Schraeder, 2006). SUDEP is thought to involve autonomic dysfunction which is tightly regulated by the HPA axis (Ulrich-Lai and Herman, 2009). Here we demonstrate that HPA axis dysfunction increases SUDEP incidence. Our data link HPA axis dysfunction to SUDEP risk for the first time, providing a potential novel mechanism contributing to SUDEP and opening avenues for mechanistic research into SUDEP pathophysiology.

Psychiatric illnesses are linked to sudden death unrelated to epilepsy. Psychiatric illnesses are associated with increased morbidity due to numerous factors, including suicide, comorbid alcohol and substance use, and accidents. However, individuals with psychiatric illnesses are also at an increased risk of cardiac sudden death (Uchida and Suzuki, 2015). Depression is associated with an increased risk of cardiovascular disease, coronary heart disease, and cardiac death (Musselman et al., 1998). The link between psychiatric illnesses and cardiovascular disease has been linked to environment and lifestyle, such as body weight, smoking, and lack of exercise (Nielsen et al., 2021). However, the exact biological mechanisms mediating the association between psychiatric illnesses and cardiovascular disease are unresolved (Musselman et al., 1998). Relevant to the current study, the HPA axis has also been suggested to mediate the cardiac problems and sudden death (Musselman et al., 1998), and recently, psychiatric comorbidities associated with epilepsy have been linked to increased SUDEP in PWE (Tao et al., 2021).

Emerging evidence, including findings presented in this study, demonstrate a link between stress, psychiatric illnesses, sudden death, epilepsy, and SUDEP. Stress activates the HPA axis and is a trigger for psychiatric illnesses. In fact, HPA hyperexcitability is a hallmark feature of depression. Our previous research linked HPA axis dysfunction to comorbid psychiatric illnesses and epilepsy, and here we demonstrate a novel mechanistic link to SUDEP. Stress, psychiatric illnesses, and epilepsy have all been linked to cardiovascular disease. Given that the HPA axis influences autonomic and cardiovascular function, future studies will need to examine the mechanistic link between HPA axis dysfunction, autonomic and cardiovascular function, and SUDEP.

These findings demonstrate that the *Kcc2*/*Crh* KO mouse model is a novel model of SUDEP with utility in investigating nongenetic mechanisms contributing to SUDEP. While some genetic risk factors in PWE increase susceptibility to SUDEP (Bagnall et al., 2017; Coll et al., 2019), nongenetic risk factors influencing SUDEP incidence are relatively understudied. Our model suggests that HPA axis dysfunction may be a contributing factor to increased SUDEP risk, suggesting a potential environmental link (stress) to SUDEP risk. A prominent hypothesis in the field is that SUDEP results from cardiac and/or respiratory dysfunction in PWE. Studies have shown that seizures can compromise both cardiac and respiratory (Devinsky, 2004) function in PWE. Combined with our previous work showing that seizures alone can activate the HPA axis (O'Toole et al., 2014) and work from others showing that increased HPA axis function independent of seizures contributes to compromised heart function (Pimenta et al., 2012), we propose that increased HPA axis dysfunction in chronic epilepsy can contribute to cardiorespiratory deficits that can predispose PWE to SUDEP. The provocative findings of the current manuscript open up novel avenues of research to investigate previously unexplored mechanisms contributing to SUDEP risk.

## Data Availability

The data analysis code is available on GitHub. *Kcc2*/*Crh* KO mice are available upon request. Raw data used in this study are available from the lead contact upon reasonable request. All custom Python scripts for analysis and visualization are available from the lead contact upon reasonable request. Mobility-mapper for behavioral scoring is available at https://github.com/researchgrant/mobility-mapper. Seizure detection app is available at https://github.com/neurosimata/seizy.

## Synthesis

Reviewing Editor: Harald Sontheimer, University of Virginia Decisions are customarily a result of the Reviewing Editor and the peer reviewers coming together and discussing their recommendations until a consensus is reached. When revisions are invited, a fact-based synthesis statement explaining their decision and outlining what is needed to prepare a revision will be listed below. The following reviewer(s) agreed to reveal their identity: NONE.

Both referees feel that this is very innovative work, well executed and will move the field forward considerably. Both offered some suggestions for improvements which I am listing unedited below. I ask you to look at these carefully and if you agree make changes accordingly. Note that these are all considered minor issues.

Referee 1:

> it was not apparent to me why only male mice were studied in figure 1, especially considering how every other experiment in the study is performed on both males and females. do the authors have data on females they can include? if not, can they justify why it is not necessary?

> would the authors consider a revised title? the most significant findings of the paper are sex-specific, and it seems a bit of a missed opportunity to not include this in the title...maybe something along the lines of "Hypothalamic-pituitary-adrenal axis dysfunction elevates SUDEP risk in a sex-specific manner"

in general the authors did a very nice job of showing both means via bar graph and individual sample values with dots. however, this is missing from figure 1 and should be added

> it was a bit confusing at first to read about the one male mouse that died of SUDEP in the +CNO condition as being in the week when CO is removed (lines 243-244). would it be possible to add a visual representation of the +CNO and -CNO to the experimental timeline in the main body of the figure. since the methods section is so abbreviated and almost entirely in supplement, it would be nice if there was an easy way to communicate to the reader when those mice were on and off CNO without having to go to the supplemental methods

> could the authors please provide one or more citations for the claim in lines 347-349. I think this is a very interesting and some readers will want to read more about this, but currently there are no citations to point the reader to find more literature on this topic

> the discussion section includes a nice discussion of male/female differences in co-morbidities with epilepsy as well as a nice discussion of stress and SUDEP. however, unless I missed it, there is little to no discussion of sex-differences in seizure burden or SUDEP

> "We confirmed that the increased risk of SUDEP in this model is directly related to

HPA axis dysfunction since chemogenetic or pharmacological suppression of the HPA signaling prevents SUDEP incidence in this model" the authors ought to consider rephrase this as the statistical test for the chemogenetic suppression was not significant

Referee 2

My only recommendations for improvement are minor clarifications and one questionable minor point in the discussion.

122 The fact that the tables are supplementary was unclear from the text.

126-130 The fact that Figure 2 panel I is in extended data is not clear from the main text. It is not clear to this reviewer that much is gained by fitting smoothed functions to the distributions rather than simply saying the means or numbers of animals on either side of a threshold were different. If the plots are to be included it would be helpful to also plot the unsmoothed histograms (or individual data points) so that it is easier to tell how well sampled these distributions are.

298: Presumably the upward ticks in the survival curves of Figure 6 are times at which animals were sacrificed. It would be clearer to state this in the legend. In addition it is hard to relate the pie charts to the survival curves since the Kcc2/Crh data are replotted, but this is not immediately obvious because the timescales are different. It would help if the replotting (of survival curves but not of pie charts) was stated in the legend.

359: typo: "Although have reduced cortisol..."

360: "...there is" → "...there are"

367 "cortisol levels are stable over the time collection" This point seems a bit questionable since the study cited measured salivary cortisol, but the measurements here are from blood.

390-91: It might be clearer to change: "psychiatric comorbidities associated with epilepsy have been linked to SUDEP" to "increased SUDEP in PWE..." or "increased SUDEP in epilepsy patients..." "have been linked to psychiatric comorbidities."

## References

[B1] Bagnall RD, Crompton DE, Semsarian C (2017) Genetic basis of sudden unexpected death in epilepsy. Front Neurol 8:348. 10.3389/fneur.2017.00348 28775708 PMC5517398

[B2] Brandt C, Schoendienst M, Trentowska M, May TW, Pohlmann-Eden B, Tuschen-Caffier B, Schrecke M, Fueratsch N, Witte-Boelt K, Ebner A (2010) Prevalence of anxiety disorders in patients with refractory focal epilepsy—a prospective clinic based survey. Epilepsy Behav 17:259–263. 10.1016/j.yebeh.2009.12.009 20075009

[B3] Coll M, Oliva A, Grassi S, Brugada R, Campuzano O (2019) Update on the genetic basis of sudden unexpected death in epilepsy. Int J Mol Sci 20:1979. 10.3390/ijms20081979 31018519 PMC6515014

[B4] Colmers PLW, Antonoudiou P, Basu T, Scapa G, Fuller P, Maguire J (2023) Loss of PV interneurons in the BLA contributes to altered network and behavioral states in chronically epileptic mice. bioRxiv, 2023.2012.2005.570112. 10.1523/ENEURO.0482-23.2024 PMC11773627 39746805

[B5] de Kloet ER, Joels M, Holsboer F (2005) Stress and the brain: from adaptation to disease. Nat Rev Neurosci 6:463–475. 10.1038/nrn1683 15891777

[B6] Devinsky O (2004) Effects of seizures on autonomic and cardiovascular function. Epilepsy Curr 4:43–46. 10.1111/j.1535-7597.2004.42001.x 15562299 PMC531654

[B7] Devinsky O, Hesdorffer DC, Thurman DJ, Lhatoo S, Richerson G (2016) Sudden unexpected death in epilepsy: epidemiology, mechanisms, and prevention. Lancet Neurol 15:1075–1088. 10.1016/S1474-4422(16)30158-2 27571159

[B8] Dorninger F, Zeitler G, Berger J (2020) Nestlet shredding and nest building tests to assess features of psychiatric disorders in mice. Bio Protocol 10:e3863. 10.21769/BioProtoc.3863 33473360 PMC7116606

[B9] Garde AH, Hansen AM (2005) Long-term stability of salivary cortisol. Scand J Clin Lab Invest 65:433–436. 10.1080/00365510510025773 16081365

[B10] Gaus V, Kiep H, Holtkamp M, Burkert S, Kendel F (2015) Gender differences in depression, but not in anxiety in people with epilepsy. Seizure 32:37–42. 10.1016/j.seizure.2015.07.012 26552559

[B11] Ginsberg JP (2016) Editorial: dysregulation of autonomic cardiac control by traumatic stress and anxiety. Front Psychol 7:945. 10.3389/fpsyg.2016.00945 27445913 PMC4914824

[B12] Gröticke I, Hoffmann K, Loscher W (2007) Behavioral alterations in the pilocarpine model of temporal lobe epilepsy in mice. Exp Neurol 207:329–349. 10.1016/j.expneurol.2007.06.021 17714705

[B13] Gröticke I, Hoffmann K, Löscher W (2008) Behavioral alterations in a mouse model of temporal lobe epilepsy induced by intrahippocampal injection of kainate. Exp Neurol 213:71–83. 10.1016/j.expneurol.2008.04.036 18585709

[B14] Hesdorffer DC, et al. (2011) Combined analysis of risk factors for SUDEP. Epilepsia 52:1150–1159. 10.1111/j.1528-1167.2010.02952.x 21671925

[B15] Hooper A, Paracha R, Maguire J (2018) Seizure-induced activation of the HPA axis increases seizure frequency and comorbid depression-like behaviors. Epilepsy Behav 78:124–133. 10.1016/j.yebeh.2017.10.025 29186699 PMC7847314

[B16] Kanner AM (2003) Depression in epilepsy: prevalence, clinical semiology, pathogenic mechanisms, and treatment. Biol Psychiatry 54:388–398. 10.1016/S0006-3223(03)00469-4 12893113

[B17] Kanner AM (2006) Depression and epilepsy: a new perspective on two closely related disorders. Epilepsy Curr 6:141–146. 10.1111/j.1535-7511.2006.00125.x 17260039 PMC1783479

[B18] Krugers HJ, Lucassen PJ, Karst H, Joels M (2010) Chronic stress effects on hippocampal structure and synaptic function: relevance for depression and normalization by anti-glucocorticoid treatment. Front Synaptic Neurosci 2:24. 10.3389/fnsyn.2010.00024 21423510 PMC3059694

[B19] Lampert R (2009) Emotion and sudden cardiac death. Expert Rev Cardiovasc Ther 7:723–725. 10.1586/erc.09.75 19589107

[B20] Lathers CM, Schraeder PL (2006) Stress and sudden death. Epilepsy Behav 9:236–242. 10.1016/j.yebeh.2006.06.001 16872908

[B21] Li X, Morrow D, Witkin JM (2006) Decreases in nestlet shredding of mice by serotonin uptake inhibitors: comparison with marble burying. Life Sci 78:1933–1939. 10.1016/j.lfs.2005.08.002 16182315

[B22] McEwen BS (2016) Stress-induced remodeling of hippocampal CA3 pyramidal neurons. Brain Res 1645:50–54. 10.1016/j.brainres.2015.12.043 26740399

[B23] McEwen BS, Magarinos AM (2017) Stress effects on morphology and function of the hippocampus. Ann N Y Acad Sci 821:271–284. 10.1111/j.1749-6632.1997.tb48286.x 9238211

[B24] McEwen BS, Nasca C, Gray JD (2016) Stress effects on neuronal structure: hippocampus, amygdala, and prefrontal cortex. Neuropsychopharmacology 41:3. 10.1038/npp.2015.171 26076834 PMC4677120

[B25] Melon LC, Hooper A, Yang X, Moss SJ, Maguire J (2018) Inability to suppress the stress-induced activation of the HPA axis during the peripartum period engenders deficits in postpartum behaviors in mice. Psychoneuroendocrinology 90:182–193. 10.1016/j.psyneuen.2017.12.003 29274662 PMC6613202

[B26] Muller CJ, Gröticke I, Bankstahl M, Loscher W (2009) Behavioral and cognitive alterations, spontaneous seizures, and neuropathology developing after a pilocarpine-induced status epilepticus in C57BL/6 mice. Exp Neurol 219:284–297. 10.1016/j.expneurol.2009.05.035 19500573

[B27] Musselman DL, Evans DL, Nemeroff CB (1998) The relationship of depression to cardiovascular disease: epidemiology, biology, and treatment. Arch Gen Psychiatry 55:580–592. 10.1001/archpsyc.55.7.580 9672048

[B28] Nielsen RE, Banner J, Jensen SE (2021) Cardiovascular disease in patients with severe mental illness. Nat Rev Cardiol 18:136–145. 10.1038/s41569-020-00463-7 33128044

[B29] O'Toole KK, Hooper A, Wakefield S, Maguire J (2014) Seizure-induced disinhibition of the HPA axis increases seizure susceptibility. Epilepsy Res 108:29–43. 10.1016/j.eplepsyres.2013.10.013 24225328 PMC3872265

[B30] Palmiter RD (2008) Dopamine signaling in the dorsal striatum is essential for motivated behaviors: lessons from dopamine-deficient mice. Ann N Y Acad Sci 1129:35–46. 10.1196/annals.1417.003 18591467 PMC2720267

[B31] Pariante CM, Lightman SL (2008) The HPA axis in major depression: classical theories and new developments. Trends Neurosci 31:464–468. 10.1016/j.tins.2008.06.006 18675469

[B32] Pimenta E, Wolley M, Stowasser M (2012) Adverse cardiovascular outcomes of corticosteroid excess. Endocrinology 153:5137–5142. 10.1210/en.2012-1573 22919065

[B33] Revdal E, Kolstad BP, Winsvold BS, Selmer KK, Morken G, Brodtkorb E (2023) Psychiatric comorbidity in relation to clinical characteristics of epilepsy: a retrospective observational study. Seizure 110:136–143. 10.1016/j.seizure.2023.06.011 37379699

[B34] Sawyer NT, Escayg A (2010) Stress and epilepsy: multiple models, multiple outcomes. J Clin Neurophysiol 27:445–452. 10.1097/WNP.0b013e3181fe0573 21076337

[B35] Sveinsson O, Andersson T, Carlsson S, Tomson T (2017) The incidence of SUDEP. Neurology 89:170–177. 10.1212/WNL.0000000000004094 28592455

[B36] Tao G, et al. (2021) Association between psychiatric comorbidities and mortality in epilepsy. Neurol Clin Pract 11:429–437. 10.1212/CPJ.0000000000001114 34824893 PMC8610550

[B37] Uchida H, Suzuki T (2015) Cardiac sudden death in psychiatric patients. Can J Psychiatry 60:203–205. 10.1177/070674371506000501 26174522 PMC4484688

[B38] Ulrich-Lai YM, Herman JP (2009) Neural regulation of endocrine and autonomic stress responses. Nat Rev Neurosci 10:397–409. 10.1038/nrn2647 19469025 PMC4240627

[B39] Vlastelica M (2008) Emotional stress as a trigger in sudden cardiac death. Psychiatr Danub 20:411–414. 18827773

[B40] Wulsin AC, Kraus KL, Gaitonde KD, Suru V, Arafa SR, Packard BA, Herman JP, Danzer SC (2021) The glucocorticoid receptor specific modulator CORT108297 reduces brain pathology following status epilepticus. Exp Neurol 341:113703. 10.1016/j.expneurol.2021.113703 33745919 PMC8169587

[B41] Wulsin AC, Solomon MB, Privitera MD, Danzer SC, Herman JP (2016) Hypothalamic-pituitary-adrenocortical axis dysfunction in epilepsy. Physiol Behav 166:22–31. 10.1016/j.physbeh.2016.05.015 27195458 PMC5053854

[B42] Zeidler Z, Brandt-Fontaine M, Leintz C, Krook-Magnuson C, Netoff T, Krook-Magnuson E (2018) Targeting the mouse ventral hippocampus in the intrahippocampal kainic acid model of temporal lobe epilepsy. eNeuro 5:ENEURO.0158-18.2018. 10.1523/ENEURO.0158-18.2018 30131968 PMC6102375

[B43] Zhu X-R, Zhu Z-R, Wang L-X, Zhao T, Han X (2022) Prevalence and risk factors for depression and anxiety in adult patients with epilepsy: caregivers’ anxiety and place of residence do mater. Epilepsy Behav 129:108628. 10.1016/j.yebeh.2022.108628 35245762

