# Supplementary figures and images for: Hypothalamic–Pituitary–Adrenal Axis Dysfunction Elevates SUDEP Risk in a Sex-Specific Manner
Source: eNeuro. 2024 Jul 9;11(7):ENEURO.0162-24.2024. doi: 10.1523/ENEURO.0162-24.2024 (PMC11236591; doi:10.1523/ENEURO.0162-24.2024)

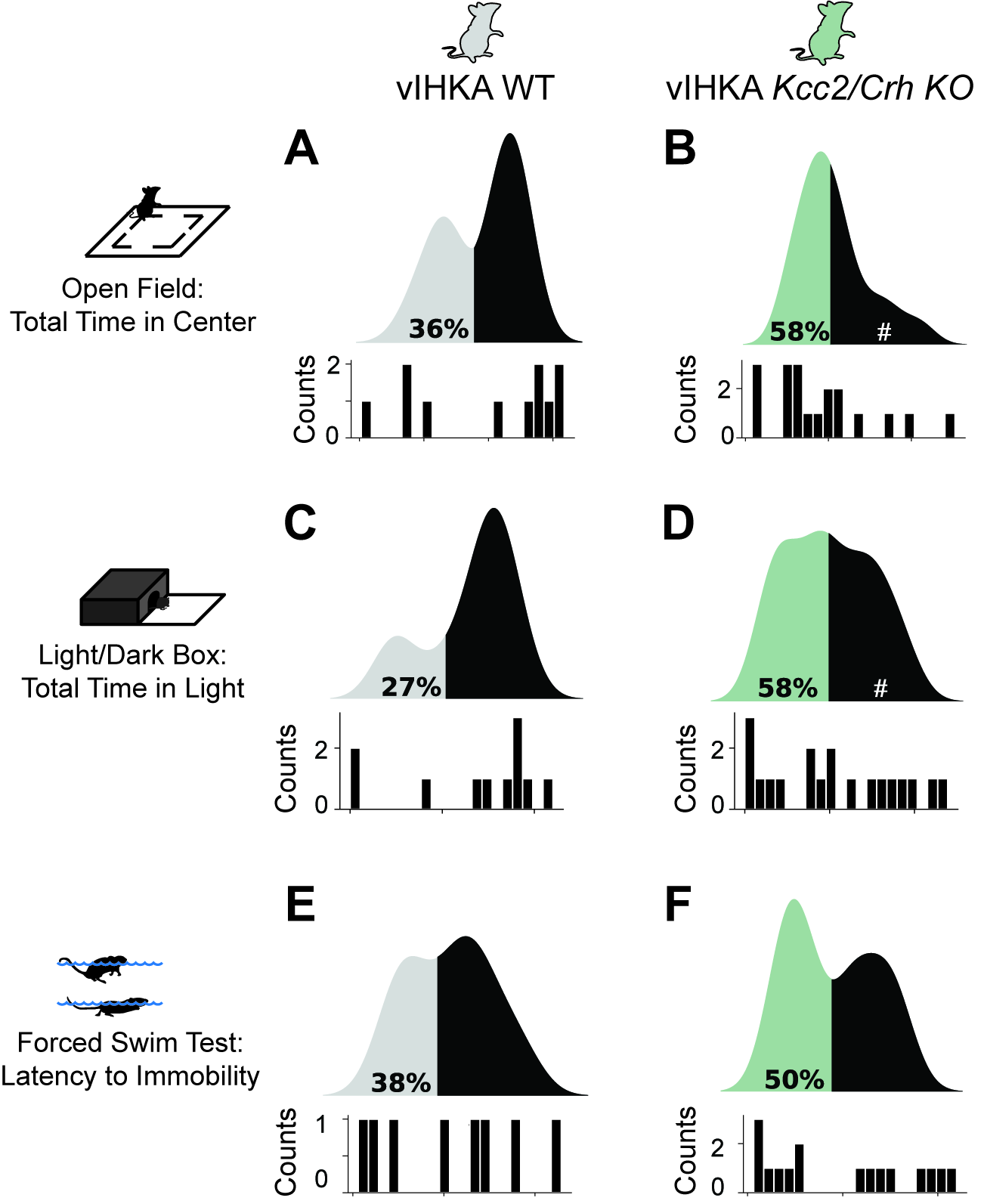

Supplement: Figure 2-1 — A greater proportion of the chronically epileptic Kcc2/Crh KO mouse population exhibit increased vulnerability to negative affective states compared to chronically epileptic (vIHKA) WT mice. Smoothed population distributions (top) along with raw histogram distribution (bottom) of performance in the Open Field (A-B), Light Dark box (C-D), and Forced Swim Test (E-F) between chronically epileptic WT (left) and Kcc2/Crh KO (right) mice. In each smoothed plot, the lighter color represents the underperforming, more vulnerable population while the black distribution plots represent the resilient groups. # denotes instances where only one peak was detected, so population distributions were delineated by the mean of the data. Download Figure 2-1, TIF file. [file eneuro-11-ENEURO.0162-24.2024-s001.tif]

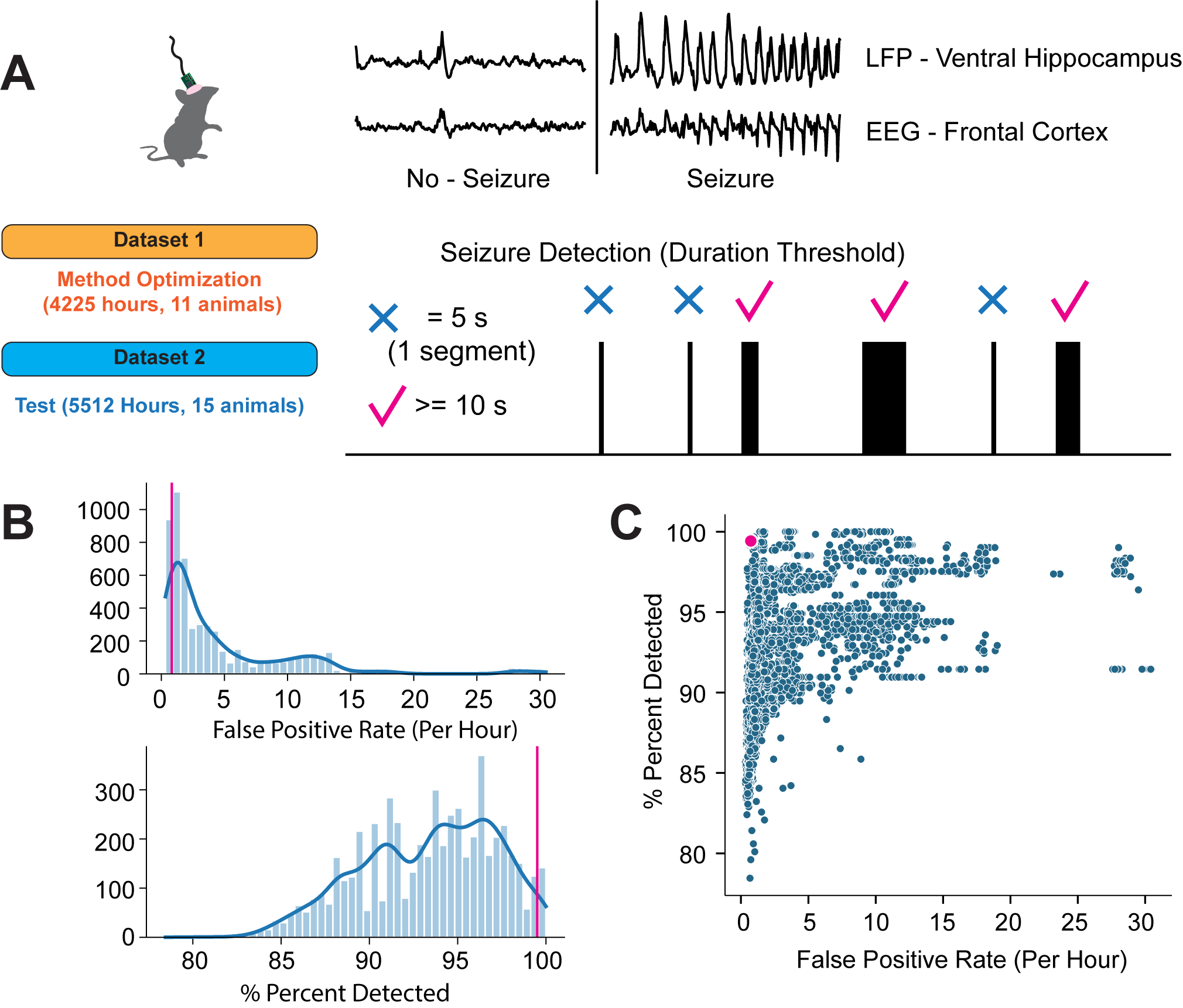

Supplement: Figure 4-1 — Seizure detection pipeline. (A) Left - Datasets used to train and test the seizure detection algorithm, Upper Right – Example traces for no-seizure and seizure (5 second periods), Bottom Right – Minimum number of seizure segments for an event to qualify as a seizure. (B) Histograms with KDE plots showing the Top – number of false detected seizures per hour and Bottom – percentage of detected seizures; Pink line denotes performance of chosen method. (C) Scatterplot of percent detected seizures vs false positive rate. Pink dot indicates performance of chosen method. Detailed pipelines can be found at https://github.com/neurosimata/seizy. Download Figure 4-1, TIF file. [file eneuro-11-ENEURO.0162-24.2024-s002.tif]

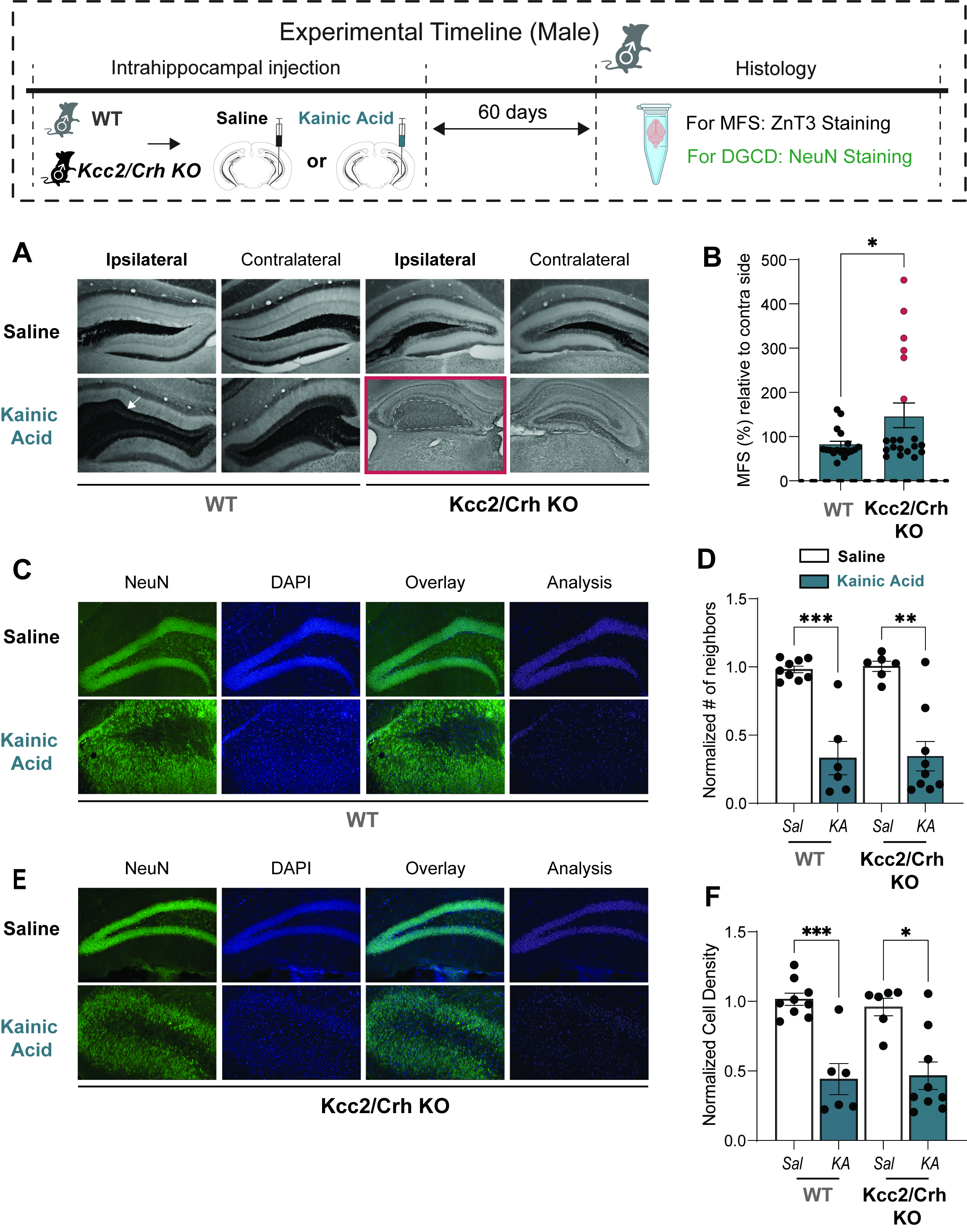

Supplement: Figure 4-2 — HPA axis dysfunction worsens MFS in male chronically epileptic mice. (A) Representative coronal sections of the hippocampus collected from control and chronically epileptic adult, male WT and Kcc2/Crh KO mice and stained with ZnT3 to quantify MFS. White arrow indicates mossy fiber sprouting. In some slices, we observed that dentate completely lost structural integrity as assessed by ZnT3 staining; example indicated by a red outline (B) The mean (±SEM) percent change in MFS was quantified in the ipsilateral hemisphere and normalized to the mean percent change in MFS of the contralateral hemisphere of chronically epileptic WT and Kcc2/Crh KO mice. Dotted black line indicates no MFS in animals that received saline injection. In slices with complete loss of structural integrity, MFS was quantified as the full dentate length. Those slices are indicated by colored filled dots on the graph. (C-D) Representative sections stained with NeuN to visualize DGCD in WT (C) and Kcc2/Crh mice (D). Pink outlines were automatically generated through Cell Profiler and indicate cells where NeuN and DAPI colocalize. (E) The mean (±SEM) number of adjoining neighboring neuronal cells was quantified for the ipsilateral hemisphere and normalized to the mean number of immediate neighboring neuronal cells on the contralateral hemisphere for both control and chronically epileptic WT and Kcc2/Crh KO mice. (F) The total number of cells within the manually defined dentate gyrus area was quantified on the ipsilateral hippocampal hemisphere and normalized to the cell density of the non-injected, contralateral hippocampal hemisphere. n = brain slice sections. Error bars represent ± SEM. WT, wild type; Sal, saline; KA, kainic acid; DGCD, dentate granule cell dispersion; Norm, normalized. Download Figure 4-2, TIF file. [file eneuro-11-ENEURO.0162-24.2024-s003.tif]

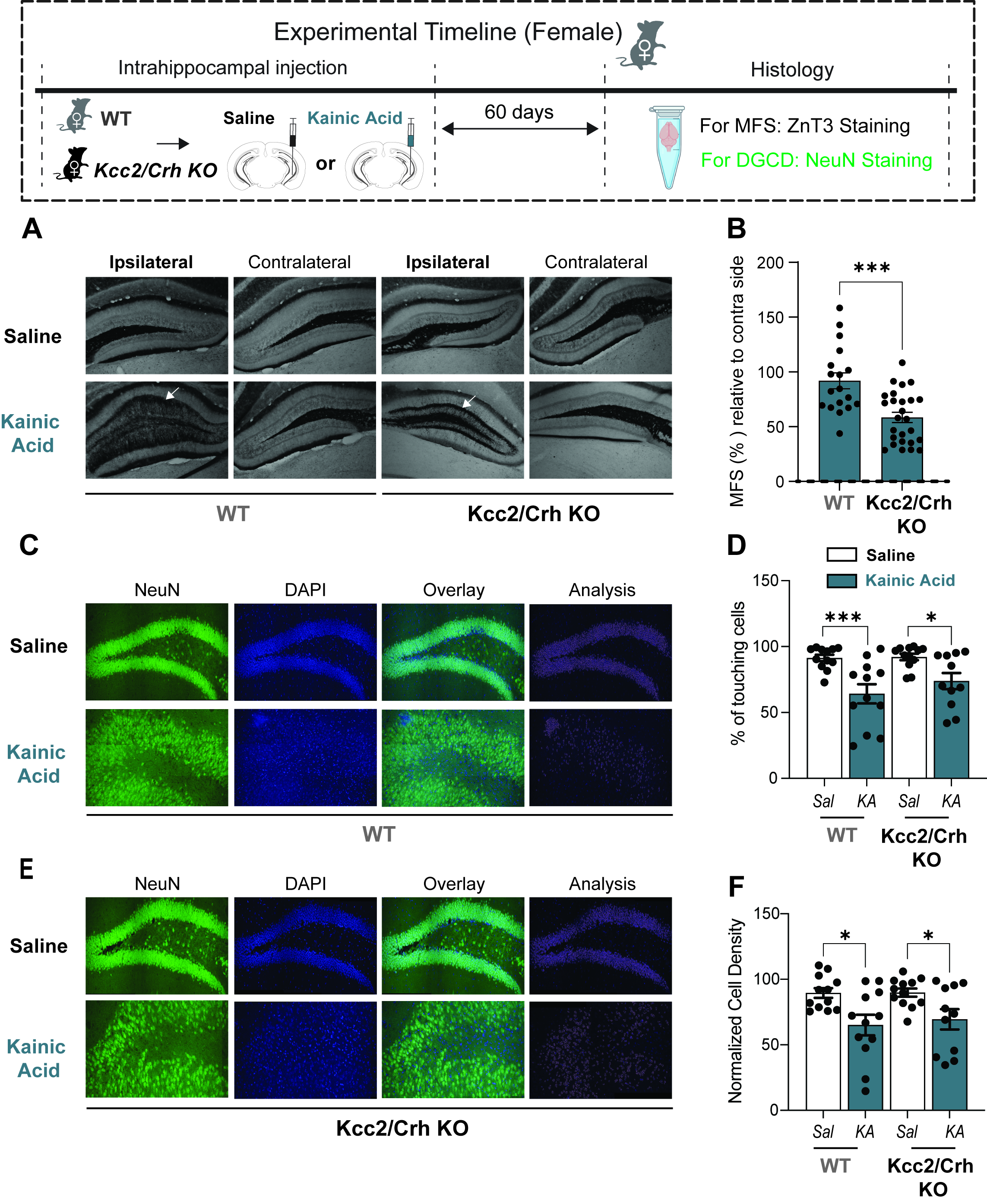

Supplement: Figure 5-1 — HPA axis dysfunction worsens MFS in female chronically epileptic mice. (A) Representative coronal sections of the hippocampus collected from control and chronically epileptic adult, female WT and Kcc2/Crh KO mice and stained with ZnT3 to quantify MFS. White arrows indicate MFS. (B) The mean (±SEM) percent change in MFS was quantified in the ipsilateral hemisphere and normalized to the mean percent change in MFS of the contralateral hemisphere of chronically epileptic WT and Kcc2/Crh KO mice. Dotted black line indicates no MFS in animals that received saline injection. Representative sections stained with NeuN to visualize DGCD in WT (C) and Kcc2/Crh KO mice (D). Pink outlines were automatically generated through Cell Profiler and indicate cells where NeuN and DAPI colocalize. (E) The mean (±SEM) number of adjoining neighboring neuronal cells was quantified for the ipsilateral hemisphere and normalized to the mean number of immediate neighboring neuronal cells on the contralateral hemisphere for both control and chronically epileptic WT and Kcc2/Crh KO female mice. (F) The total number of cells within the manually defined dentate gyrus area was quantified on the ipsilateral hippocampal hemisphere and normalized to the cell density of the non-injected, contralateral hippocampal hemisphere. n = brain slice sections. Error bars represent ± SEM. WT, wild type; Sal, saline; KA, kainic acid; DGCD, dentate granule cell dispersion; Norm, normalized. Download Figure 5-1, TIF file. [file eneuro-11-ENEURO.0162-24.2024-s004.tif]
